# Labor and birth care by nurse with midwifery skills in Brazil

**DOI:** 10.1186/s12978-016-0236-7

**Published:** 2016-10-17

**Authors:** Silvana Granado Nogueira da Gama, Elaine Fernandes Viellas, Jacqueline Alves Torres, Maria Helena Bastos, Odaléa Maria Brüggemann, Mariza Miranda Theme Filha, Arthur Orlando Correa Schilithz, Maria do Carmo Leal

**Affiliations:** 1 National School of Public Health, Oswaldo Cruz Foundation (ENSP/Fiocruz), Rio de Janeiro, Brazil; 2 National Agency of Supplemental Health (ANS), Rio de Janeiro, Brazil; 3 Federal University of Santa Catarina (UFSC), Santa Catarina, Brazil; 4 ESCOLA NACIONAL DE SAÚDE PÚBLICA SERGIO AROUCA - ENSP/FIOCRUZ, Rua Leopoldo Bulhões, 1480 - Manguinhos, Rio de Janeiro, CEP: 21041-210 Brazil; 5 National Cancer Institute (INCA), Rio de Janeiro, Brazil

**Keywords:** Maternal and child health, Obstetric labour, Good practices in obstetrics, Vaginal birth, Nurse-midwife, Midwife, Midwifery

## Abstract

**Background:**

The participation of nurses and midwives in vaginal birth care is limited in Brazil, and there are no national data regarding their involvement. The goal was to describe the participation of nurses and nurse-midwives in childbirth care in Brazil in the years 2011 and 2012, and to analyze the association between hospitals with nurses and nurse-midwives in labor and birth care and the use of good practices, and their influence in the reduction of unnecessary interventions, including cesarean sections.

**Methods:**

Birth in Brazil is a national, population-based study consisting of 23,894 postpartum women, carried out in the period between February 2011 and October 2012, in 266 healthcare settings. The study included all vaginal births involving physicians or nurses/nurse-midwives. A logistic regression model was used to examine the association between the implementation of good practices and suitable interventions during labor and birth, and whether care was a physician or a nurse/nurse-midwife led care. We developed another model to assess the association between the use of obstetric interventions during labor and birth to the personnel responsible for the care of the patient, comparing hospitals with decisions revolving exclusively around a physician to those that also included nurses/nurse-midwives as responsible for vaginal births.

**Results:**

16.2 % of vaginal births were assisted by a nurse/nurse-midwife. Good practices were significantly more frequent in those births assisted by nurses/nurse-midwives (*ad lib.* diet, mobility during labor, non-pharmacological means of pain relief, and use of a partograph), while some interventions were less frequently used (anesthesia, lithotomy position, uterine fundal pressure and episiotomy). In maternity wards that included a nurse/nurse-midwife in labour and birth care, the incidence of cesarean section was lower.

**Conclusions:**

The results of this study illustrate the potential benefit of collaborative work between physicians and nurses/nurse-midwives in labor and birth care. The adoption of good practices in managing labor and birth could be the first step toward more effective obstetric and midwifery care in Brazil. It may be easier to introduce new approaches rather than to eliminate old ones, which may explain why the reduction of unnecessary interventions during labor and birth was less pronounced than the adoption of new practices.

**Electronic supplementary material:**

The online version of this article (doi:10.1186/s12978-016-0236-7) contains supplementary material, which is available to authorized users.

## Background

The role of the midwife in supporting care during pregnancy, birth, and the postpartum period is well established in many countries. Currently, the World Health Organization and the United Nations Population Fund recommend the leadership and involvement of a midwife or a nurse with midwifery skills in prenatal care as well as for the management of labor and vaginal birth [1, 2].

Studies in *The Lancet* Midwifery Series [3, 4] regarding the quality of obstetric and neonatal care show that midwifery, especially when offered by a midwife in collaboration with physicians and other team members, is safe and effective in the reduction of maternal and neonatal mortality.

For decades, childbirth care in Brazil was administered by traditional birth attendants with participation of midwives, who received specific training from the Faculty of Medicine. In the mid-twentieth century nurses began to assume this role, and gradually traditional birth attendants and trained by doctors were replaced [5]. The practice of nursing with obstetric skills was regulated by decree in 1961 for those who held a qualification or a specialization certificate in obstetrics [6].

From 1972, nurses with obstetric skills could only obtain their degree in nursing schools and, apart from the obstetricians, they were the sole professionals qualified to assist labor and birth. Since 1986, the role of nurses with midwifery skills has been supported by the law of professional practice, number 7498/86 [6].

In order to implement a new model of care for women’s health in Brazil, in the year 2000 the Ministry of Health started to fund post-graduate courses in nursing and midwifery across the country both as a residency/specialization and as further training for nurses who already worked in maternity care [7]. These courses emphasize “humanizing” practices that take place during the labor and birth process, with the objectives of avoiding unnecessary interventions and safeguarding the privacy and autonomy of women during pregnancy, labor, birth and the postpartum period [8, 9].

This measure recognized the importance of nursing and midwifery in implementing a new policy for women’s health [7, 8], reducing the activities of nurses without midwifery skills in labor and birth care.

In 2005, was launched the first direct-entry graduate course in midwifery in Brazil, with an innovative curriculum inspired by successful international experiences. In 2007, the Brazilian Association of Midwives and Nurses-midwives – ABENFO officially recognized the Essential Competencies for Basic Midwifery Practice proposed by the International Confederation of Midwives – ICM as a standard to define essential skills and behaviors required for safe midwifery practice in any setting. In line with this, in 2014, the Pan American Health Organization - PAHO adapted for the region the Strengthening Midwifery Toolkit. This document is a guide for midwifery training in Brazil and considers that nurse-midwives and midwives should be qualified to support the physical, emotional and socio-cultural needs of women, in the family and in the community context, especially during pregnancy, childbirth and the postpartum period to guarantee the main goals of The World Health Organization for the Safe Motherhood Initiative [10].

Nevertheless, the participation of nurses and nurse-midwives in vaginal birth care in Brazil is limited, and there are no national data regarding their involvement, except for some local studies in a few maternity hospitals [11–13].

The goals of this study are twofold. Firstly, this study aims to describe the participation of nurses/nurse-midwives in caring for vaginal births in Brazil between 2011 and 2012. Secondly, to analyze the association between hospitals with nurses/nurse-midwives in labour and birth care and the appropriate use of good practices, including the reduction of unnecessary interventions such as cesarean section.

## Methods

Birth in Brazil was a national hospital-based study of postpartum women and their newborns carried out between February 2011 and October 2012. We acquired the samples in three stages. The first sample consisted of hospitals with 500 or more births per year, stratified by the five national macro-regions, location (capital or non-capital), and type of hospital (private, public, or both). In the second stage of sampling, we used an inverse sampling method to select as many days as necessary to reach 90 postnatal women interviewed in the hospital (minimum of 7 days for each hospital), and the third sample was comprised of the postpartum women themselves. In each of the 266 hospitals that were included we interviewed 90 postpartum women, totaling 23,894 subjects. More information about the sampling design can be found in Vasconcellos et al. [14]. In the first phase of the study, we conducted face-to-face interviews with postpartum women during their stay at the hospital, and data were extracted from the medical charts for the women and their newborns, with photographs taken of their prenatal care records [15].

Data for this article came from the hospital interviews and women’s postpartum medical charts. We took into account the characteristics of women, and of the practices and interventions implemented during labor and birth, for all vaginal births cared for by physicians or nurses/nurse-midwives, regardless of training in midwifery (*N* = 11,499).

**Fig. 1:**
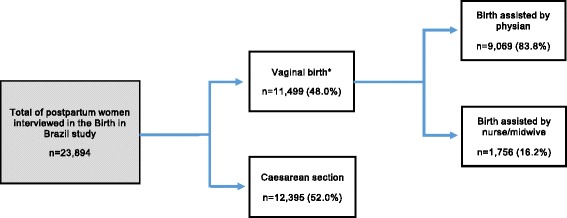
Description of vaginal birth covered in the study, according to the type of professional Brazil, 2011-2012. * 674 childbirths attended by other professionals (traditional midwife, student, nursing professional (technical, assistant) and others) or without information

In the Birth in Brazil study there were no midwives graduated from a direct-entry course in midwifery working in maternity wards, due to the fact the course was only recently established in the country [13]. Therefore, in the article we refer to nurse-midwives the nurses with midwifery skills, who, upon graduating, proceeded to undergo specialization course in midwifery. It is also possible that outside of the large urban areas, some nursing professionals who assist births have not had specialized midwifery training, although theoretical and practical training in obstetrics is part of nursing undergraduate curriculum. Alongside the article we refer to nurses/nurse-midwives to the combination of both professionals: nurses with midwifery skills and a small parcel of nursing professionals with no specialized midwifery training. The selection of vaginal births considered in this study, according to the health professional (physician or nurse/nurse-midwife) that assisted in each birth, is shown in Fig. 1.

For the analysis of labor and birth care techniques (good practices and obstetric interventions) according to the maternity hospital where the birth occurred, we considered hospitals where nurses/nurse-midwives provided the health care as be the unit of analysis, regardless of having specific training in midwifery.

In terms of categorizing the care received by women, we examined the following exposure variables: geographical region in Brazil (North, Northeast, Southeast, South, or Central); location (capital or non-capital); and whether birth care received was part of the public or private healthcare system. Maternal characteristics included age bracket (<20, 20–34, or 35 years or over); self-reported skin color/race based on the five categories used by the Brazilian Institute of Geography and Statistics (IBGE: white, black, brown, yellow, or indigenous); marital status (with or without partner); socioeconomic status based on the criteria of the Brazilian Association of Research Companies – ABIPEME [16] (A/B, C, or D/E); years of education (<8, 8–11, or ≥ 12 years); parity - number of previous births (nulliparous, 1–2 or ≥3 births); and obstetric risk (high or low).

Women considered as having low obstetric risk were those with no gestational or pre-gestational diabetes or hypertension, who were not obese, were HIV negative and had a confirmed singleton pregnancy, whose birth was vaginal, cephalic and at term (37–41° weeks gestation). Birthweight had to be in the range of 2,500 to 4,499 grams and the 5th to 95th percentiles for gestational age. We included neonatal factors as proxies for obstetric risk in order to exclude mothers with pathologies that were not omitted by the foregoing criteria [17].

Women who gave birth in public facilities, or in private ones with public funding, were classified as “public payment” and women whose births were self-funded, or paid for by a private health insurance, were classified as “private payment”.

We considered the following to be good practices in the care of labor and birth: free intake of liquids and solids (*ad lib.* diet), ambulation/mobility during the first stage of labor, the use of non-pharmacological means of pain relief, and monitoring of labor progress using a partograph. We examined interventions that took place during labor as follows: use of a peripheral venous catheter, use of oxytocin to accelerate labor, amniotomy (artificial rupture of membranes), and spinal/epidural analgesia. Similarly, those interventions assessed that took place during birth were as follows: lithotomy position, use of the uterine fundal pressure maneuver (Kristeller), and episiotomy. With respect to interventions carried out during labor and birth, cesarean section was subject solely to multivariate analysis of the examined care techniques in order to gauge the benefit of a nurse/nurse-midwife participating in childbirth care.

We applied Pearson’s chi-squared test to compare characteristics of the women according to the provider who cared for each birth. A logistic regression model was then used to relate the use of good practices and interventions during labor and birth to who oversaw the process (physician or nurse/nurse-midwife). We developed another model to assess the association between the use of assistive techniques during labor and birth to the personnel responsible for the care of the patient, comparing healthcare settings with decisions revolving exclusively around a physician to those that included the participation of a nurse/nurse-midwife in childbirth care.

Given the study design, we set the level of significance at 5 %, and odds ratios (OR) were calculated. We adjusted the ORs for region, location, source of funding, years of education, and parity. The software used for analysis was SPSS 20.0 and Microsoft Excel (2007).

This study was approved by the Ethics Committee of the Sergio Arouca National School of Public Health, Oswaldo Cruz Foundation (ENSP/Fiocruz), protocol number 92/10. All hospital directors and postpartum women signed an informed consent form before the interviews confirming their willingness to take part and answer any questions posed.

## Results

Of the 23,894 births, 48 % were vaginal births, totaling 11,499 postpartum women and their newborns. Of these, 16.2 % were cared for by nurses/nurse-midwives (Fig. 1).

The distribution by geographical region revealed a greater proportion of births with the participation of a nurse/nurse-midwife in the North (24.1 %) and the Southeast (23.5 %), with the lowest proportion in the Central Region (<1.0 %). There was no difference according to locality (capital or non-capital). With respect to funding for childbirth care, nurse/nurse-midwife participation was lower in the private sector (Table 1).

**Table 1:** Frequency of births according to type of provider who assisted in each birth. Brazil, 2011–2012

		Provider who assisted in each birth (*N* = 11,499)		*p*-value*
		Physicians (%)	Nurses/nurse-midwives (%)	
Geographical region	North	75.9	24.1	0.001
	Northeast	90.1	9.9	
	Southeast	76.5	23.5	
	South	92.5	7.5	
	Central	99.2	0.8	
Location	Non-capital	83.4	16.6	0.877
	Capital	84.3	15.7	
Source of payment	Public	83.5	16.5	0.054
	Private	92.2	7.8	

*p-value of chi-square tests of comparison between physicians and nurses/midwives

Physicians tended to care for a larger number of adolescents, without differences according to skin color or marital status. Accordingly, the distribution was roughly equivalent in terms of years of education and economic class, suggesting a certain socioeconomic equivalence between the two groups.

As for parity, nurses/nurse-midwives assisted fewer births in primiparous women compared with physicians. In addition, the proportion of high-risk pregnancies was almost equal between physicians and nurses/nurse-midwives (35.7 % and 36.1 %, respectively) (Table 2).

**Table 2:** Characteristics of postpartum women according to type of provider who assisted in each birth. Brazil, 2011–2012

		Provider who assisted in each birth (*N* = 11,499)		*p*-value*
		Physicians (%)	Nurses/nurse-midwives (%)	
Age (years)	12 to 19	25.5	22.4	0.040
	20 to 34	66.8	70.0	
	≥35	7.7	7.6	
Self-reported skin color/race	White	27.5	26.8	0.261
	Black	10.5	8.3	
	Brown	60.2	63.8	
	Yellow	1.1	0.9	
	Indigenous	0.7	0.2	
Marital status	With partner	79.1	77.2	0.243
	Without partner	20.9	22.8	
Socioeconomic status	A/B	14.7	13.2	0.592
	C	54.9	57.6	
	D/E	30.4	29.2	
Years of education	<8	34.2	32.9	0.362
	8 to 11	30.0	30.5	
	≥12	35.8	36.6	
Parity	Nulliparous	43.6	39.0	0.002
	1–2 births	43.6	44.8	
	≥3 births	12.8	16.2	
Obstetric risk	Low	64.3	63.9	0.854
	High	35.7	36.1	

* *p*-value of chi-square tests of comparison between physicians and nurses/midwives

As shown in Table 3, the *ad lib.* diet was used 2.35 times more for women under the care of a nurse/nurse-midwife, compared with those cared for by physicians. Mobility during labor occurred in only half of the cases, but was more frequent for women attended by a nurse/nurse-midwife (OR = 1.74).

**Table 3:** Adjusted ORs for the use of good practices and obstetric interventions during labor e birth according to type of provider who assisted in each birth. Brazil, 2011–2012

	Provider who assisted in each birth (*N* = 11,499)			OR^a^	
	Physicians (%)	Nurses/nurse-midwives (%)	Total (%)		95 % CI
Best practices during labor					
Free intake of liquids and solids (*ad lib.* diet)	26.1	48.7	29.8	2.35	1.62–3.39
Mobility during labor	47.9	61.1	50.1	1.74	1.29–2.34
Use of nonpharmacological pain relief	28.7	45.1	31.3	1.87	1.29–2.72
Monitoring progress of labor using a partograph	51.9	68.3	54.6	1.94	1.15–3.29
Interventions during labor					
Peripheral venous catheter	72.3	64.7	71.1	0.66	0.43–1.03
Oxytocin drip	47.2	47.3	47.2	0.90	0.59–1.37
Amniotomy	53.9	50.6	53.3	0.83	0.57–1.21
Spinal/epidural analgesia	11.1	4.8	10.1	0.29	0.12–0.72
Interventions during birth					
Lithotomy position	92.8	87.5	92.0	0.44	0.25–0.77
Uterine fundal pressure (Kristeller)	38.7	27.2	36.8	0.56	0.41–0.76
Episiotomy	57.7	38.9	54.6	0.42	0.26–0.67

*95 % CI* 95 % confidence interval, *OR* odds ratio

^a^Model adjusted for geographical region, location, age, years of education, source payment and parity

A third of women received non-pharmacological pain relief during labour (31.3 %), with a greater uptake in women whose births were nurse-assisted (OR = 1.87). The use of a partograph, which is recommended for monitoring and recording the progress of labour, was limited (54.6 %); however, it was nearly two times more likely to be employed by a nurse/nurse-midwife. Placement of a peripheral venous catheter continues to be a routine practice for both physicians (72.3 %) and nurses/nurse-midwives (64.7 %). The use of oxytocin during labor and the practice of artificial rupture of membranes were applied for about half of the women, regardless of whether care was provided by physicians or nurses/nurse-midwives. Lithotomy position predominated at the time of birth, at 92 %, but was less frequent in women cared for by a nurse/nurse-midwife (OR = 0.44, 95 % CI: 0.25–0.77).

The prevalence of Kristeller maneuver and episiotomy for vaginal births were very high, 36.8 % and 54.6 %, respectively. However, both interventions were significantly less frequent for births assisted by nurses/nurse-midwives than for the ones assisted by physicians - OR = 0.56; CI 95 % 0.41–0.76 and OR = 0.42; CI 95 % 0.26–0.67, respectively.

Of the 266 sites studied, less than one-third (*N* = 84) had births attended by a nurse/nurse-midwife during the data collection period. In the multivariate analysis (Table 4), it was observed that in the maternity ward with a nurse conducting labor and birth care, there was a greater chance of the implementation of good practices and a reduction in the use of obstetric interventions.

**Table 4:** Adjusted OR for the use of good practices and obstetric interventions during labor and birth according maternity that included the participation of a nurse or nurse-midwife during birth care. Brazil, 2011–2012

	Maternity that included the participation of a nurse/nurse-midwive during birth care (*N* = 84)		
	Total (%)	OR^a^	95 % CI
Best practices during labor			
Free intake of liquids and solids (*ad lib.* diet)	33.9	2.24	1.61–3.12
Mobility during labor	57.0	1.73	1.32–2.27
Use of nonpharmacological pain relief	34.3	2.09	1.57–2.79
Monitoring progress of labor using a partograph	49.0	1.85	1.15–2.96
Interventions during labor			
Peripheral venous catheter	67.5	0.74	0.54–1.03
Oxytocin drip	44.1	0.93	0.69–1.24
Amniotomy	49.9	0.70	0.52–0.94
Spinal/epidural analgesia	4.7	0.36	0.14–0.91
Interventions during birth			
Lithotomy position	92.6	1.04	0.48–2.23
Uterine fundal pressure (Kristeller)	33.6	0.65	0.51–0.82
Episiotomy	47.1	0.54	0.37–0.79
Caesarian section	41.4	0.78	0.62–0.98

*95 % CI* 95 % confidence interval, *OR* odds ratio

^a^Model adjusted for geographical region, location, age, years of education, source payment and parity

In these maternity wards, the *ad lib.* diet was 2.24 times more common compared with those without nurse/nurse-midwife participation in labor and birth care. The likelihood of mobility during labor was 73 % greater, the chance of a woman being offered non-pharmacological methods of pain relief was more than two times higher, and the use of a partograph was 85 % higher. On the other hand, we observed significantly lower odds for amniotomy (OR = 0.70), spinal/epidural analgesia (OR = 0.36), Kristeller maneuver (OR = 0.65), and episiotomy (OR = 0.54) in women who gave birth in these facilities. In addition, the rate of cesarean section was lower in maternities that had nurses/nurse-midwives in birth care (41.4 %), while in traditional care settings the rate was 58.4 % (OR = 0.78, 95 % CI: 0.62–0.98). There was no significant statistical difference in the use of peripheral venous catheters or use of oxytocin during labor or in opting for lithotomy position during birth.

## Discussion

In Brazil, during 2011 and 2012, only 7.7 % of all births were nurse or nurse-midwife led. When considering only vaginal births, this proportion rises to 16.2 %. There was no difference in the obstetric risk profile between vaginal births cared for by physicians and those cared for by a nurse/nurse-midwife. The implementation of good practices in labor and birth care, recommended by the World Health Organization [18], was significantly more frequent in those births assisted by a nurse/nurse-midwife than in those assisted by physicians. Obstetric interventions were very common in births cared for by both types of health care providers. The presence of a nurse/nurse-midwife in the maternity care team had a positive impact, including a reduction in the rate of cesarean section.

The North and Southeast Regions saw the greatest frequency of childbirth care led by a nurse/nurse-midwife, but for different reasons. In the North, it was for a lack of physicians, whereas in the Southeast it was due to the purposeful inclusion of nurse-midwives in the childbirth care model.

The North Region is the poorest area of the country. It is a vast territory that includes the Amazon rainforest; many cities are isolated and accessible only by boat or airplane [19]. It has the lowest number of doctors per 1,000 inhabitants (1.01) [20] and the highest occurrence of home births in the country (3.96 %) [21], which tend to be cared for by traditional birth attendants.

The Southeast Region, the richest region in the country, contains the highest number of physicians per 1,000 inhabitants (2.67) [20] and the lowest rate of home births (0.22 %) [21]. Since the end of the 1990s, this region has seen the adoption of “humanizing” policies in labor and birth care, especially in the state capital cities. These policies have led to the participation of a nurse-midwife in routine practice, carrying for women with low-risk births in some public and private facilities [22–26].

We found nursing and medical care of vaginal birth to be virtually identical in terms of the demographic and socioeconomic characteristics of women, probably because of the strong correlation between social class and type of birth. In Brazil, nearly 90 % of births in the private sector, where the majority of women of a higher socioeconomic class are cared for, occur via cesarean section. Although the private sector represents approximately 20 % of births in Brazil, from the 11,499 vaginal births analyzed, only 578 (5.0 %) happened in this sector (data not shown).

As such, women included in this study were mostly cared for in the public sector, and thus are similar in terms of socioeconomic factors, such as years of education, social class and marital status. The only observed differences were maternal age and parity, with the majority of primiparous women and adolescents cared for by physicians. Other national studies have also found that a lower proportion of primiparous births are managed by a nurse/nurse-midwife [27].

Nurses/nurse-midwives, as well as physicians, exposed women to excessive interventions. Despite strict guidelines for oxytocin administration in the induction or augmentation of labour, nearly half of all women received the drug, suggesting a tendency to routinely use the substance in isolation or in combination with other procedures. Such routine use should be avoided, as it increases the difficulty for women’s mobility in labour and because of the related side effects, such as uterine tachysystole, hypertonic uterine dysfunction, uterine rupture and acute fetal distress [28–30]. Similarly, episiotomy and placement of a venous catheter for hydration as routine support have not been proved beneficial for women [31–33]. Even though a woman’s birthing position should be her choice and respected by the care team [34], the majority of women gave birth in lithotomy position, most times with someone performing Kristeller maneuver; such practices can cause discomfort, pain, and pose risks for women, their newborn and have subsequently been banned in many countries [35].

Overall, nurses/nurse-midwives facilitate greater use of good practices in labor and birth. In a study carried out in Minas Gerais, Brazil, in two facilities participating in the National Health System (known as SUS), it was found that inclusion of a nurse-midwife in a collaborative care team was linked with less frequent use of oxytocin for augmentation of labor, lower rates of artificial rupture of membranes and episiotomy, and greater use of non-pharmacological pain relief during labor [36]. Even in Birth Centers (health facilities linked to a hospital for low-risk birth care, physiological puerperium and care of healthy newborn) where nurse-midwives have autonomy over care practices, the use of oxytocin was still high, varying between 24 % [37] and 31 % [38].

In Rio de Janeiro, studies examining the public sector’s uptake of “humanizing” policies found that nurses/nurse-midwives do incorporate the corresponding practices and appropriate communication in labor and birth care. However, in order to assert themselves in a field traditionally dominated by physicians, they comply with the prevailing technical model, not directly resisting the use of interventions but gradually reducing their use and integrating practices for “humanizing” care [25, 39].

One finding that stood out in this study was the presence of a nurse or a nurse-midwife in labor and birth care helped to reduce cesarean section rates for a given facility. This study has found similar results to another study of an innovative private hospital, which had 76 % of its births assisted by nurse-midwives and a cesarean section rate of 47 % - nearly half of what has been estimated for the Brazilian private sector, where physicians control obstetric care [26].

The strength of this study is that we have used a representative nationwide survey, with primary data collected from medical records. This allowed, for the first time, a description of the participation of nurses/nurse-midwives assisting vaginal births and their positive influence in implementing good practices and appropriate interventions during labor and birth in Brazil.

One limitation of this study is that only hospitals with more than 500 births per year were eligible for the Birth in Brazil study, leading to the exclusion of those with fewer births that apparently are more frequent in cities far from the large urban centers. As such, we were not able to evaluate the involvement of the nurse/nurse-midwife during labor and birth care in those areas, which account for 20 % of all national births.

Another limitation is that the inclusion of a nursing professional in labor and birth care is materializing slowly in Brazil, and for this reason we chose to examine the participation of all nurses, regardless of specific training in midwifery. However, although the vast majority of nurses providing labour and birth care in Brazil have been trained with some midwifery skills, it was not possible to distinguish the proportion of births attended by professionals with this training in accordance with ICM competency standards.

## Conclusion

The results of this study illustrate the potential benefit of collaborative work between physicians and nurses/nurse-midwives in labor and birth care. The adoption of good practices in managing labor and birth could be the first step toward more effective childbirth care in Brazil. It is likely that it will be easier to introduce new approaches rather than to eliminate routine practices. This may explain why the reduction of unnecessary interventions during labor and birth was less pronounced than the adoption of new practices.

In this study the lower cesarean section rates may be the result of greater participation on the part of the nurse/nurse-midwife, with a more equitable distribution of responsibilities among members of the care team. Such distribution of tasks will allow physicians to focus their attention on those cases that require obstetric intervention. Furthermore, increased application of the aforementioned good practices in labor and birth care may have empowered women to participate more fully in determining their labor and birth care, thus enabling nurses/nurse-midwives and physicians to share their knowledge, which in turn was reflected in the institution’s care model [40].

Our findings show that the model of health care advocated by the Brazilian Ministry of Health, which focuses on policies for the humanization of maternity care, the increase in the use of good practices, promoting privacy, advocating respect and autonomy of women’s choices, and the reduction of unnecessary interventions during labor and birth, is expanding visibility where nurse-midwives and midwives are leading care in assisting vaginal births in Brazil.

## Additional files

Additional file 1: Portuguese version. (DOC 271 kb)
Additional file 2: Peer review. (DOC 57 kb)
