# Supplementary material for: Labor and birth care by nurse with midwifery skills in Brazil
Source: Reprod Health. 2016 Oct 17;13(Suppl 3):123. doi: 10.1186/s12978-016-0236-7 (PMC5073910; doi:10.1186/s12978-016-0236-7)
Supplement: Additional file 1: — Portuguese version. (DOC 271 kb) [file 12978_2016_236_MOESM1_ESM.doc]

**Assistência ao trabalho de parto e parto por enfermeiras no Brasil**

**Resumo**

**Introdução:** A participação de enfermeiras e enfermeiras-obstétricas na assistência ao parto vaginal no Brasil é restrita, e não há dados nacionais que quantifiquem seu envolvimento. O objetivo foi descrever a participação de enfermeiras na assistência ao parto vaginal no Brasil, nos anos de 2011 e 2012, e analisar a associação entre hospitais com enfermeiras na assistência direta ao parto e o uso de boas práticas no trabalho de parto e parto, e a redução de intervenções desnecessárias, inclusive de cesarianas.

**Métodos**: Nascer no Brasil é um estudo nacional, de base populacional consistido por 23.894 mulheres no pós-parto, realizado no período compreendido entre fevereiro de 2011 e outubro de 2012, em 266 serviços de saúde. O estudo incluiu todos os partos vaginais envolvendo médicos ou enfermeiras. Um modelo de regressão logística foi utilizado para identificar associação entre a implementação de boas práticas e intervenções durante o trabalho de parto e parto, segundo o profissional que atendeu à mulher, médico ou enfermeira. Foi desenvolvido outro modelo para avaliar a associação entre o uso de boas práticas na assistência durante o trabalho de parto e parto, comparando hospitais com ou sem enfermeiras responsáveis pela assistência ao parto vaginal.

**Resultados:** 16,2% dos partos vaginais foram assistidos por enfermeira. As boas práticas obstétricas foram significativamente mais frequentes nos partos assistidos por enfermeira (*ad lib.* dieta, mobilidade durante o trabalho, métodos não farmacológicos de alívio da dor e uso de um partograma), enquanto algumas intervenções foram menos utilizadas por essas profissionais (anestesia, posição de litotomia, manobra de Kristeller e episiotomia). A incidência de cesariana foi menor nas maternidades que incluíam enfermeira na assistência ao parto e nascimento.

**Conclusões**: Os resultados deste estudo ilustram o potencial benefício do trabalho colaborativo entre médicos e enfermeiras no trabalho de parto e assistência ao parto. A adoção de boas práticas no manejo do trabalho e parto pode ser o primeiro passo em direção a cuidados obstétricos mais efetivos no Brasil. Ficou claro ser mais fácil introduzir novas abordagens do que eliminar as antigas, o que pode explicar porque a redução das intervenções desnecessárias foi menos pronunciada do que a adoção de novas práticas.

**Palavras-chaves**: Saúde materno-infantil, Trabalho de parto, Boas práticas em obstetrícia, Parto vaginal, Enfermeira-obstétrica, Obstetriz, Obstetrícia

**Introdução**

O papel da enfermeira-obstétrica e obstetriz na assistência à gestação, parto e puerpério está bem estabelecido em muitos países. Atualmente estes profissionais são recomendados pela Organização Mundial de Saúde (OMS) e Fundo para População e Desenvolvimento das Nações Unidas (UNFPA) por terem competência suficiente para a atenção pré-natal, manejo do trabalho de parto e parto vaginal [1,2]

Estudos da Série *The Lancet Midwifery* [3,4] sobre a qualidade da atenção obstétrica e neonatal mostram que a prática de partejar, especialmente quando ofertada por enfermeira-obstétrica ou obstetriz, em trabalho colaborativo com médicos e demais membros da equipe, é segura e efetiva para redução da mortalidade materna e neonatal.

Durante décadas, a assistência ao parto no Brasil foi conduzida por parteiras tradicionais com a participação de obstetrizes, que recebiam formação específica da Faculdade de Medicina. Em meados do século XX, as enfermeiras começaram a assumir esse papel e as parteiras tradicionais e obstetrizes formadas por médicos foram gradualmente sendo substituídas [5]. A prática da enfermagem com habilidades obstétricas foi regulamentada por decreto em 1961 para aquelas que realizavam qualificação ou especialização em obstetrícia [6].

A partir de 1972 passa a ser, exclusivamente, das escolas de enfermagem a tarefa de formar enfermeiras-obstétricas que, além das obstetrizes, passam a ser os únicos profissionais não-médicos habilitados para assistir ao parto e nascimento. Desde 1986 a atuação da enfermeira-obstétrica para a assistência ao parto vaginal se torna amparada pela lei do exercício profissional de número 7498/86 [6].

Visando a implementação de um “Novo Modelo de Atenção à Saúde da Mulher no Brasil”, nos anos 2000, o Ministério da Saúde passou a financiar cursos de pós-graduação *lato sensu* em obstetrícia, em todo o país, tanto na modalidade de residência quanto de especialização, e formação complementar para as enfermeiras que já atuavam na atenção ao parto em maternidades [7]. Estes cursos enfatizam práticas de "humanização" ao longo do processo de parto e nascimento, com objetivos de evitar intervenções desnecessárias e salvaguardar a privacidade e autonomia das mulheres durante a gravidez, trabalho de parto, parto e pós-parto [8,9].

Com essa medida, reconheceu-se a importância da enfermeira-obstetra e obstetriz na implementação da Política de Saúde da Mulher no país [7,8], promovendo assim a redução de enfermeiras não habilitadas na assistência ao parto e nascimento.

Em 2005, foi lançado o primeiro curso de graduação em obstetrícia no Brasil, com um currículo inovador e inspirado em experiências internacionais bem-sucedidas. Em 2007, a Associação Brasileira de Obstetrizes e Enfermeiros Obstetras - ABENFO reconheceu oficialmente as competências básicas essenciais para a prática de obstetrícia proposta por *The International Confederation of Midwives* – ICM como um padrão para definir habilidades e comportamentos essenciais necessários à prática obstétrica segura em qualquer ambiente. Em consonância, em 2014, a Organização Pan-Americana da Saúde - OPAS adaptou para a região o documento “*The Strengthening Midwifery Toolkit*”. Este documento é uma referência para a formação em obstetrícia no Brasil, e considera que enfermeiras obstétricas e obstetrizes devem ser qualificados para lidar com as necessidades físicas, emocionais e socioculturais das mulheres, na família e no contexto da comunidade, especialmente durante a gravidez, o parto e o período pós-parto para garantir os principais objetivos da Iniciativa Maternidade Segura da Organização Mundial da Saúde [10].

No entanto, a participação de enfermeiras e enfermeiras-obstétricas na assistência ao parto vaginal no Brasil é limitado e não existem dados nacionais sobre o seu envolvimento, com exceção de alguns estudos locais em algumas maternidades [11–13].

Esse artigo teve dois objetivos: descrever a participação de enfermeiras na assistência ao parto vaginal no Brasil em comparação aos médicos, nos anos de 2011 e 2012, e analisar a associação entre hospitais com e sem enfermeiras na assistência direta ao parto, o uso de boas práticas no trabalho de parto e parto, e a redução de intervenções desnecessárias, inclusive de cesarianas.

**Metodologia**

A pesquisa *Nascer no Brasil* foi um estudo nacional de base populacional composto por puérperas e seus recém-nascidos, realizado no período de fevereiro de 2011 a outubro de 2012. A amostra foi selecionada em três estágios. O primeiro, composto por hospitais com 500 ou mais partos/ano estratificado pelas cinco macrorregiões do país, localização (capital ou não capital), e tipo de hospital (privado, público e misto). Na segunda etapa da amostragem, foi utilizado um método de amostragem inversa para selecionar quantos dias seriam necessários para atingir 90 mulheres a serem entrevistadas no hospital (mínimo de sete dias em cada hospital) e o terceiro composto pelas puérperas. Em cada um dos 266 hospitais amostrados foram entrevistados 90 puérperas, totalizando 23.894 sujeitos. Mais informações sobre o desenho amostral encontram-se detalhadas em Vasconcellos et al [14]. Na primeira fase do estudo foram realizadas entrevistas face a face com as puérperas durante a internação hospitalar e extraídos dados do prontuário da puérpera e do recém-nato, e fotografados os cartões de pré-natal [15].

Neste artigo, foram utilizados dados da entrevista hospitalar e de prontuário das puérperas. Para descrição das características das mulheres, das boas práticas e das intervenções durante o trabalho de parto e parto foram incluídos todos os nascimentos de parto vaginal, assistidos por profissional médico ou por enfermeiro, independentemente da especialidade em obstetrícia (N=11.499).

No período do estudo *Nascer no Brasil* não havia obstetrizes na atenção ao parto nas maternidades amostradas, devido ao fato de o curso só recentemente ter formado sua primeira turma no país [13]. Portanto, no artigo, referem-se a enfermeiras tanto àquelas com habilidades obstétricas, que, após se formar, passam por um curso de especialização em obstetrícia quanto profissionais de enfermagem que assistem partos sem formação especializada em obstetrícia, embora seja oferecida disciplina teórica e prática em obstetrícia como parte do currículo de graduação em enfermagem. Assim, nesse artigo, referem-se enfermeiras como combinação dos dois profissionais, sabendo que é pequena a parcela dos profissionais de enfermagem sem formação em obstetrícia atuando na atenção direta ao parto. A descrição dos partos vaginais abordados no estudo, segundo o tipo de profissional de assistência está representada na Figura 1, a seguir:

(Figura 1)

Para a análise específica do uso de boas práticas e intervenções obstétricas, segundo a maternidade de realização do parto, foram considerados como unidade de análise os hospitais (com parto vaginal assistido por enfermeira ou não, independente de sua especialização em obstetrícia).

Em relação aos serviços, as variáveis de exposição estudadas foram: região do Brasil (Norte, Nordeste, Sudeste, Sul, Centro-Oeste); localização (Capital ou Não capital); fonte de pagamento do parto (pública ou privada). Características maternas: faixa etária (< 20 anos; 20-34 anos; 35 anos ou mais); cor da pele autorrelatada, segundo as categorias utilizadas pelo Instituto Brasileiro de Geografia e Estatística - IBGE (branca, preta, parda, amarela, indígena); situação conjugal (com ou sem companheiro); classe econômica pelo critério da Associação Brasileira de Empresas de Pesquisa – ABIPEME [16] (A/B, C ou D/E); anos de estudo (<8 anos, 8-11 anos, 12 anos ou mais); número de partos anteriores (nulípara, 1 a 2 partos ou ≥ 3 partos); e risco obstétrico (alto ou baixo risco).

Foram definidas como mulheres de baixo risco obstétrico aquelas sem história de diabe­tes ou hipertensão gestacional ou pré-gestacional, não obesas, HIV negativas, com gestação de feto único e nascido de parto vaginal, em apresen­tação cefálica, a termo (37 a 41 semanas gestacionais), com peso ao nascer entre 2.500g e 4.499g e entre o 5º e 95º percentil de peso ao nascer por idade gestacional. Os fatores neonatais foram adicionados por terem sido considerados uma *proxy* do baixo risco obstétrico, sendo capazes de excluir gestantes com outras patologias não incluídas no critério [17].

Mulheres com parto em unidades públicas ou privadas financiadas pelo setor público foram classificadas como “fonte de pagamento pública”. Mulheres com parto pago por plano de saúde ou desembolso direto foram classificadas como “fonte de pagamento privada”.

Foram consideradas boas práticas durante o trabalho de parto e parto: ingestão de líquidos e alimentos (dieta livre), mobilidade durante o primeiro estágio do trabalho de parto (deambulação), uso de métodos não farmacológicos para alívio da dor e monitoramento do progresso do trabalho de parto pelo partograma. Foram consideradas como intervenções durante o trabalho de parto: uso de cateter venoso, uso de ocitocina para aceleração do trabalho de parto, amniotomia (ruptura artificial de membranas), analgesia raquidiana/epidural; e como intervenções durante o parto: posição de litotomia, manobra de Kristeller e episiotomia. Em relação às intervenções durante o parto e nascimento, a cesariana foi analisada somente na análise multivariada das associações do uso de tecnologias na assistência ao parto em maternidades, com ou sem, enfermeiras assistindo partos.

O teste de qui-quadrado de Pearson foi utilizado na comparação das proporções das características das mulheres segundo o profissional que assistiu o parto. Modelo de regressão logística foi empregado para relacionar o uso de boas práticas e intervenções durante o trabalho de parto e parto segundo o tipo de profissional que prestou a assistência, médico ou enfermeira/enfermeira-obstétrica. Outro modelo foi elaborado para testar a associação entre o uso de tecnologias na assistência ao parto e nascimento, comparando as unidades de saúde com assistência centrada exclusivamente no médico e aquela com a participação direta de enfermeira/enfermeira-obstétrica na assistência ao parto.

O nível de significância estabelecido foi de 5% e utilizaram-se as estimativas de razão de chances (OR) considerando o efeito do desenho amostral. As OR foram ajustadas para região, capital e não capital, fonte de pagamento para o parto, idade materna, escolaridade e paridade. O software empregado nas análises foi o SPSS 20.0 e Microsoft Excel versão 2007.

O estudo foi aprovado pelo Comitê de Ética em Pesquisa da Escola Nacional de Saúde Pública Sergio Arouca, Fundação Oswaldo Cruz (ENSP/Fiocruz), parecer nº 92/10 e todas as puérperas assinaram o termo de consentimento livre e esclarecido antes da entrevista.

**Resultados**

Do total de 23.894 partos analisados, 48% foram vaginais, totalizando 11.499 puérperas e seus conceptos. A assistência foi de responsabilidade da enfermeira em 16,2% dos casos (Figura 1).

A distribuição por regiões geográficas do país revelou maiores proporções de partos realizados por enfermeiras nas Regiões Norte (24,1%) e Sudeste (23,5%), sendo a menor proporção na Região Centro-Oeste (<1,0%). Não foram observadas diferenças segundo profissional que atendeu ao parto ocorridos nas capitais ou demais cidades. Em relação à fonte de financiamento, a participação dos enfermeiros foi menor na rede privada (Tabela 1).

Os médicos atenderam proporcionalmente mais puérperas adolescentes, não se observando diferenças em relação à cor da pele e situação conjugal. Do mesmo modo, a distribuição foi homogênea em relação ao nível de escolaridade e classe econômica das mulheres atendidas, o que mostra certo equilíbrio socioeconômico entre os dois grupos.

Quanto à paridade, enfermeiras assistiram menos partos de primíparas em comparação aos médicos, e a proporção de partos com risco foi equivalente entre médicos e enfermeiras, 35,7% e 36,1%, respectivamente (Tabela 2).

Como exposto na Tabela 3, a dieta livre foi 2,35 vezes maior nas mulheres assistidas por enfermeiras quando comparadas aos médicos. A deambulação no pré-parto ocorreu em apenas metade das mulheres, porém mais frequente naquelas assistidas por enfermeiras (OR=1,74).

Ainda são pouco utilizados os métodos não farmacológicos para alívio da dor no trabalho de parto (31,3%), sendo o uso maior em mulheres cujo parto foi atendido por enfermeiras (OR=1,87). O uso do partograma, recomendado para o monitoramento da evolução do trabalho de parto, foi pouco empregado (54,6%), porém, quase 2 vezes mais utilizado pelas enfermeiras.

A colocação de cateter venoso permanece como uma prática rotineira tanto por médicos (72,3%) quanto por enfermeiras (64,7%). O uso de ocitocina no trabalho de parto e a prática da amniotomia foram referidos por cerca de metade das mulheres, independentemente de que profissional prestou assistência. Prevaleceu amplamente a litotomia como posição utilizada pelas mulheres no momento do parto, chegando a 92% dos casos, ainda assim menos frequente quando atendidos por enfermeiras (OR=0,44; IC 95% 0,25-0,77).

A manobra de Kristeller (36,8%) e a episiotomia (54,6%) foram bastante frequentes nos partos vaginais, sendo significativamente menos empregados por enfermeiras (OR=0,56; IC95% 0,41-0,76 e OR=0,42; IC95% 0,26-0,67, respectivamente).

Dos 266 estabelecimentos estudados, menos de um terço (N=84) tiveram pelo menos um parto assistido por enfermeiro entre as 90 puérperas entrevistadas. Na análise multivariada (Tabela 4) observa-se que, em maternidades onde enfermeiros assistem diretamente ao parto, existe mais chance de realização das boas práticas e redução do uso de intervenções obstétricas.

Nessas maternidades a dieta livre foi 2,24 vezes maior quando comparada às maternidades sem enfermeiras na assistência ao parto. A chance de deambulação no pré-parto foi 73% maior, a oferta de métodos não farmacológicos para aliviar as dores no pré-parto foi mais de duas vezes e o uso de partograma foi 85% maior. Por outro lado, foi significativamente menor a chance de amniotomia (OR=0,70), anestesia (OR=0,36), manobra de Kristeller (OR=0,65) e episiotomia (OR = 0,54) nas mulheres que pariram nesses estabelecimentos. Inclusive a proporção de cesariana foi menor nas maternidades que adotam enfermeiras como responsáveis pela atenção ao parto (41,4%), enquanto nos modelos tradicionais foi 58,4% (OR=0,78; IC 95% 0,62-0,98). Não houve diferença estatisticamente significativa em relação ao uso de cateter venoso e ocitocina no trabalho de parto, e da posição de litotomia no momento do parto.

**Discussão**

No Brasil, nos anos de 2011 e 2012, apenas 7,7% do total de nascimentos foram assistidos por enfermeiras. Considerando-se apenas os partos vaginais, a proporção aumenta para 16,2%, não havendo diferença entre o perfil de risco obstétrico nos partos vaginais assistidos por médicos e enfermeiras. A realização de boas práticas na assistência ao parto, recomendadas pela OMS [18], foi significativamente maior nos partos assistidos por enfermeiras em comparação aos médicos. As intervenções obstétricas foram muito empregadas nos partos por ambos os profissionais. A presença de enfermeiras na assistência ao parto nas equipes das maternidades teve impacto positivo inclusive na redução de cesarianas.

As Regiões Norte e Sudeste concentraram a maior proporção de partos realizados por enfermeiras. No entanto, acredita-se que na Região Norte, a ausência do profissional médico seja o principal motivo, enquanto no Sudeste isso esteja ocorrendo pela inserção da enfermeira-obstétrica no modelo de atenção ao parto.

O Norte é a região mais carente do país, tem grande dimensão territorial, com algumas cidades isoladas e de difícil acesso, apenas por barco ou avião [19]. Essa região possui a menor razão de médicos por 1.000 habitantes (1,01) [20] e as mais elevadas proporções de partos domiciliares do país (3,96%) [21], sendo a grande maioria assistido por parteiras leigas.

A Região Sudeste, a mais rica do país, possui a maior razão de médicos por 1.000 habitantes (2,67) [20] e baixa proporção de parto domiciliar (0,22%) [21]. Nessa região vêm sendo implantadas políticas de humanização do parto e nascimento desde o final da década de 1990, especialmente nas capitais dos estados, introduzindo a enfermagem obstétrica na assistência ao parto vaginal em algumas instituições públicas e privadas [22–26].

A assistência ao parto vaginal por médico ou enfermeira praticamente não se diferenciou segundo as variáveis socioeconômicas e demográficas das mulheres, provavelmente pela forte associação da classe social com o tipo de parto. No Brasil, quase 90% dos nascimentos na rede privada, onde são atendidas mulheres de classes sociais mais elevadas, ocorrem por cesariana. Embora o setor privado represente cerca de 20% dos nascimentos no Brasil, dos 11.499 partos vaginais analisados, apenas 578 (5,0%) aconteceram neste setor (dados não mostrados).

As mulheres analisadas foram majoritariamente atendidas nos serviços públicos de saúde, por isso são semelhantes socialmente. As únicas distinções observadas foram em relação à idade da puérpera e paridade, com maiores proporções de primíparas e adolescentes sob os cuidados médicos. Outros estudos nacionais também encontraram menor proporção de partos em primíparas assistidos por enfermeiras-obstétricas [27].

Tanto médicos como enfermeiras submeteram as mulheres a excessivas intervenções, cujo uso rotineiro não tem demonstrado benefícios. Apesar de inúmeras evidências que restringem o uso da ocitocina apenas para reduzir o tempo do trabalho de parto, sua utilização foi observada em quase metade das mulheres. O uso rotineiro da substância deve ser evitado uma vez que aumenta a dificuldade das mulheres para caminhar e provoca inúmeros efeitos colaterais relacionados, como taquissistolia uterina, disfunção uterina hipertônica, ruptura uterina e sofrimento fetal agudo [28–30].

Do mesmo modo, a colocação de cateter venoso para hidratação e a episiotomia como rotina da assistência não têm demonstrado benefícios para as mulheres [31–33]. E, ainda que a posição para parir deva ser de escolha da mulher e respeitada pela equipe [34], a maioria das mulheres deu à luz na posição de litotomia, muitas vezes com alguém pressionando sua barriga (manobra de Kristeller) – procedimento que causa dor e representa riscos para as mulheres e seus recém-nascidos, já banido de muitos países [35].

Contudo, enfermeiras possibilitaram maior uso das boas práticas durante o trabalho de parto e parto. Em estudo realizado em Minas Gerais – Brasil, em duas unidades de saúde conveniadas ao Sistema Único de Saúde (SUS), encontrou-se que onde havia atuação de enfermeiras-obstétricas em modelo colaborativo com o médico, houve significativamente menos utilização de ocitocina para acelerar o trabalho de parto, amniotomia*,* episiotomia e, por outro lado, foi maior o emprego de métodos não farmacológicos para alívio da dor no trabalho de parto [36]. Mesmo nos Centros de Parto Normal no Brasil, local em que enfermeiras-obstétricas possuem autonomia sobre as práticas a serem implementadas, o uso de ocitocina ainda tem sido elevado, variando de 24% [37] a 31% [38].

No Rio de Janeiro, estudos que analisaram a implantação de políticas de humanização da assistência ao parto em hospitais públicos destacaram que as enfermeiras obstétricas incorporaram os discursos e práticas humanizadas de assistência ao parto, mas, para se afirmarem em um campo tradicionalmente dominado por médicos, reproduziam o modelo tecnicista vigente, não se opondo ao uso de intervenções, embora, paulatinamente venham reduzindo seu uso e incorporando práticas de assistência humanizada [25,39].

Fato que chamou a atenção nos achados desse estudo foi a presença da enfermeira na assistência ao parto reduzir a taxa de cesariana na instituição. Resultado semelhante ao encontrado em um hospital privado inovador, no qual 76% dos partos são assistidos por enfermeiras-obstétricas e que apresentou taxa de cesariana de 47% em 2011/12, quase a metade do valor estimado para o setor privado brasileiro, onde médicos lideram o cuidado obstétrico [26].

A força deste estudo é termos uma pesquisa nacional representativa, com dados primários coletados diretamente com a puérpera e extraídos de seus prontuários. Isto permitiu, pela primeira vez, uma descrição da participação de enfermeiras na assistência ao parto vaginal e expor sua influência positiva na implementação de boas práticas e redução de intervenções durante o trabalho de parto e nascimento no Brasil.

Como limitação, para a pesquisa *Nascer no Brasil* foram elegíveis apenas hospitais com mais de 500 partos/ano, excluindo os menores, mais comuns nas cidades afastadas dos grandes centros. Assim, não temos como saber se nesses locais haveria maior ou menor inserção das enfermeiras na assistência ao parto. A soma dessas unidades totalizam 20% dos nascimentos no país.

Outra limitação é que a inserção da enfermeira/enfermeira-obstétrica na atenção ao parto ainda vem se dando lentamente no Brasil, por isso, optou-se por incluir nessa análise todas as enfermeiras, independentemente de sua especialização em obstetrícia. Ainda que saibamos que, nos últimos anos, a grande maioria das enfermeiras que prestam assistência ao parto e nascimento no país tenham especialização ou residência em obstetrícia, não foi possível distinguir a proporção de partos assistidos por profissionais com essa formação nas competências descritas pelo ICM.

**Conclusão**

Esse estudo provoca reflexões sobre o potencial do trabalho colaborativo entre médicos e enfermeiras na assistência ao parto, mostrando que a adoção de boas práticas durante o trabalho de parto e parto pode ser o primeiro passo para uma mudança mais efetiva na atenção obstétrica brasileira. É provável que seja mais fácil introduzir novas abordagens do que suprimir antigas. Isso pode explicar porque a redução de intervenções desnecessárias no trabalho de parto e parto foi menos pronunciada do que a aquisição de novas práticas.

O efeito na redução da taxa de cesariana pode ser consequência da maior delegação da atenção ao parto às enfermeiras, o que possibilita melhor compartilhamento de atribuições dentro da equipe obstétrica, direcionando a atenção médica para casos que necessitem da intervenção desse profissional. Acresce-se a isso a possibilidade de maior exposição às boas práticas, levando ao protagonismo das mulheres para a condução do seu trabalho de parto, a transferência de conhecimentos entre médicos e enfermeiras, com reflexo no modelo de atenção da instituição [40].

Os achados desse estudo expõem que o modelo de atenção à saúde da mulher, com foco nas políticas de humanização do parto e nascimento, inserção de boas práticas da atenção obstétrica, redução de intervenções desnecessárias e garantia da privacidade e autonomia das mulheres no trabalho de parto e parto tem proporcionado maior visibilidade à atuação das enfermeiras-obstétricas e obstetrizes no manejo do parto vaginal no Brasil.

**Conflito de interesse**

Os autores declaram não existir conflitos de interesse.

**Contribuições dos autores**

S. G. N. Gama e M. C. Leal participaram de todos as etapas de produção do artigo e foram responsáveis pela primeira e última versão do artigo. E. F. Viellas, J. A. Torres, M. H. Bastos, O. M. Brüggemann e M. M. Theme Filha contribuíram com a interpretação dos resultados, discussão, leitura e revisão final da versão do manuscrito. A. O. C. Schilithz contribuiu com a análise dos dados e revisão final do manuscrito.

**Acknowledgments**

Agradecemos aos coordenadores, supervisores e entrevistadores regionais e estaduais, à equipe das maternidades assim como as mulheres que participaram do estudo, tornando-o possível. Agradecemos ao Conselho Nacional de Desenvolvimento Científico e Tecnológico (CNPq), Ministério da Saúde, Escola Nacional de Saúde Pública/Fiocruz (Projeto INOVA), a Fundação de Amparo à Pesquisa do Estado do Rio de Janeiro (FAPERJ), e a Coordenação de Aperfeiçoamento de Pessoal de Nível Superior (Capes).

**Referências**

1. World Health Organization. Nursing and midwifery progress report, 2008-2012. [Internet] 2013 [28 abr 2016]. Disponível em: http://www.who.int/hrh/nursing_midwifery/NursingMidwiferyProgressReport.pdf

2. United Nations Population Fund, International Confederation of Midwives, World Health Organization, editors. The state of the world’s midwifery 2014: a universal pathway - a woman’s right to health [Internet]. New York: United Nations Population Fund; 2014. [28 abr 2016]. Disponível em: http://www.unfpa.org/sowmy

3. Renfrew MJ, McFadden A, Bastos MH, Campbell J, Channon AA, Cheung NF, et al. Midwifery and quality care: findings from a new evidence-informed framework for maternal and newborn care. The Lancet. 2014;384:1129–45.

4. Van Lerberghe W, Matthews Z, Achadi E, Ancona C, Campbell J, Channon A, et al. Country experience with strengthening of health systems and deployment of midwives in countries with high maternal mortality. The Lancet. 2014;384:1215–25.

5. Osawa RH, Riesco MLG, Tsunechiro MA. Parteiras-enfermeiras e Enfermeiras-parteiras: a interface de profissões afins, porém distintas. Rev. Bras. Enferm. 2006;59:699–702.

6. Oguisso T, Schmidt MJ, Freitas GF. Fundamentos teóricos e jurídicos da profissão de enfermagem. Enferm. Em Foco. 2010;1:9–13.

7. Costa AAN de M, Schirmer J. A atuação dos enfermeiros egressos do curso de especialização em obstetrícia no nordeste do Brasil: da proposta à operacionalização. Esc. Anna Nery. 2012;16:332–9.

8. Brasil. Portaria GM/MS n.569, de 01 de junho de 2000. Institui o Programa de Humanização no Pré-natal e Nascimento no âmbito do Sistema Único de Saúde [Internet]. Diário Oficial da União. Brasília - DF: 18 Ago 2000, p. 112. [28 abr 2016]. Disponível em: http://bvsms.saude.gov.br/bvs/saudelegis/gm/2000/prt0569_01_06_2000_rep.html

9. Pasche DF, Vilela ME de A, Martins CP. Humanização da atenção ao parto e nascimento no Brasil: pressupostos para uma nova ética na gestão e no cuidado. Rev Tempus Actas Saúde Col. 2010;4:105–17.

10. Centro Latino-Americano de Perinatologia, Saúde da Mulher e Reprodutiva. Conjunto de ferramentas para o fortalecimento da parteria nas Américas [Internet]. Montevidéu: CLAP/SMR; 2014. [28 abr 2016]. Disponível em: http://www.paho.org/clap/index.php?option=com_content&view=article&id=288%3Aconjunto-de-ferramentas-para-o-fortalecimento-da-parteria-nas-americas&catid=667%3Apublicaciones&Itemid=234&lang=en

11. Carr M, Riesco M. Rekindling of Nurse-Midwifery in Brazil: Public Policy and Childbirth Trends. J. Midwifery Womens Health. 2007;52:406–11.

12. Narchi NZ. Exercise of essential competencies for midwifery care by nurses in São Paulo, Brazil. Midwifery. 2011;27:23–9.

13. Gualda DMR, Narchi NZ, de Campos EA. Strengthening midwifery in Brazil: Education, regulation and professional association of midwives. Midwifery. 2013;29:1077–81.

14. Vasconcellos MTL de, Silva PL do N, Pereira APE, Schilithz AOC, Souza Junior PRB de, Szwarcwald CL. Desenho da amostra Nascer no Brasil: Pesquisa Nacional sobre Parto e Nascimento. Cad. Saúde Pública. 2014;30:S49–58.

15. Do Carmo Leal M, da Silva AA, Dias MA, da Gama SG, Rattner D, Moreira M, et al. Birth in Brazil: national survey into labour and birth. Reprod. Health. 2012;9:15.

16. Paiva GF dos S, Silva DB do N, Feijó CA. Consumption and Socioeconomic Classification in Brazil: A Study Based on the Brazilian Family Expenditure Survey [Internet]. 2013 [10 Jun 2015]. Disponível em: http://www.iariw.org/papers/2013/paivapaper.pdf

17. Dahlen HG, Tracy S, Tracy M, Bisits A, Brown C, Thornton C. Rates of obstetric intervention and associated perinatal mortality and morbidity among low-risk women giving birth in private and public hospitals in NSW (2000-2008): a linked data population-based cohort study. BMJ Open. 2014;4:e004551–e004551.

18. Organização Mundial de Saúde - OMS. Assistência ao parto normal: um guia prático. Saúde materna e neonatal. Unidade de maternidade segura [Internet]. Saúde Reprodutiva e da Família. Genebra (CH); 1996 [10 Jun 2015]. Disponível em: http://www.saude.mppr.mp.br/arquivos/File/kit_atencao_perinatal/manuais/assistencia_ao_parto_normal_2009.pdf

19. Paim J, Travassos C, Almeida C, Bahia L, Macinko J. The Brazilian health system: history, advances, and challenges. The Lancet. 2011;377:1778–97.

20. Scheffer M, Cassenote A, Biancarelli A. Demografia Médica no Brasil. 2013. Demografia Médica no Brasil. Cenários e indicadores de distribuição [Internet]. São Paulo: Conselho Regional de Medicina do Estado de São Paulo: Conselho Federal de Medicina; 2013 [10 Jun 2015]. Disponível em: http://www.cremesp.org.br/pdfs/DemografiaMedicaBrasilVol2.pdf

21. Brasil. MS/SVS/DASIS. Sistema de Informações sobre Nascidos Vivos - SINASC [Internet]. [10 Jun 2015]. Disponível em: http://tabnet.datasus.gov.br/cgi/deftohtm.exe?sinasc/cnv/nvuf.def

22. Dias MAB, Domingues RMSM. Desafios na implantação de uma política de humanização da assistência hospitalar ao parto. Ciênc. Saúde Coletiva. 2005;10:699–705.

23. Lobo SF, Oliveira SMJV, Schneck CA, Silva FMB da, Bonadio IC, Riesco MLG. Resultados maternos e neonatais em Centro de Parto Normal peri-hospitalar na cidade de São Paulo, Brasil. Rev. Esc. Enferm. USP. 2010;44:812–8.

24. Vogt SE, Diniz SG, Tavares CM, Santos NCP, Schneck CA, Zorzam B, et al. Características da assistência ao trabalho de parto e parto em três modelos de atenção no SUS, no Município de Belo Horizonte, Minas Gerais, Brasil. Cad. Saúde Pública. 2011;27:1789–800.

25. Progianti JM, Porfírio AB. Participation of nurses in the process of implementation of obstetrical practices in the maternity humanized Alexander Fleming (1998-2004). Esc. Anna Nery. 2012;16:443–50.

26. Torres JA, Domingues RMSM, Sandall J, Hartz Z, Gama SGN da, Filha MMT, et al. Caesarean section and neonatal outcomes in private hospitals in Brazil: comparative study of two different perinatal models of care. Cad. Saúde Pública. 2014;30:S220–31.

27. Pereira AL de F, Dantas F. Assistance characteristics of normal deliveries attended by obstetrical nurses. J. Nurs. UFPE Line. 2012;6:76–82.

28. Cecatti JG, Calderón I de MP. Intervenções benéficas durante o parto para a prevenção da mortalidade materna. Rev. Bras. Ginecol. E Obstetrícia. 2005;27:357–65.

29. Clark SL, Simpson KR, Knox GE, Garite TJ. Oxytocin: new perspectives on an old drug. Am. J. Obstet. Gynecol. 2009;200:35.e1–35.e6.

30. Bugg GJ, Siddiqui F, Thornton JG. Oxytocin versus no treatment or delayed treatment for slow progress in the first stage of spontaneous labour. In: The Cochrane Collaboration, editor. Cochrane Database Syst. Rev. [Internet]. Chichester, UK: John Wiley & Sons, Ltd; 2011 [10 Jun 2015]. Disponível em: http://doi.wiley.com/10.1002/14651858.CD007123.pub2

31. Carroli G, Mignini L. Episiotomy for vaginal birth. In: The Cochrane Collaboration, editor. Cochrane Database Syst. Rev. [Internet]. Chichester, UK: John Wiley & Sons, Ltd; 2009 [10 Jun 2015]. Disponível em: http://doi.wiley.com/10.1002/14651858.CD000081.pub2

32. Riesco MLG, Costa A de SC da, Almeida SFS de, Basile AL de O, Oliveira SMJV de. Episiotomia, laceração e integridade perineal em partos normais: análise de fatores associados. Rev Enferm UERJ. 2011;19:77–83.

33. Dawood F, Dowswell T, Quenby S. Intravenous fluids for reducing the duration of labour in low risk nulliparous women. In: The Cochrane Collaboration, editor. Cochrane Database Syst. Rev. [Internet]. Chichester, UK: John Wiley & Sons, Ltd; 2013 [10 Jun 2015]. Disponível em: http://doi.wiley.com/10.1002/14651858.CD007715.pub2

34. Brüggemann OM, Monticelli M, Furtado C, Fernandes CM, Lemos FN, Gayeski ME. Filosofia assistencial de uma maternidade-escola: fatores associados à satisfação das mulheres usuárias. Texto Contexto Enferm. 2011;20:658–68.

35. Verheijen EC, Raven JH, Hofmeyr GJ. Fundal pressure during the second stage of labour. In: The Cochrane Collaboration, editor. Cochrane Database Syst. Rev. [Internet]. Chichester, UK: John Wiley & Sons, Ltd; 2009 [10 Jun 2015]. Disponível em: http://doi.wiley.com/10.1002/14651858.CD006067.pub2

36. Vogt SE, Silva KS da, Dias MAB. Comparação de modelos de assistência ao parto em hospitais públicos. Rev. Saúde Pública. 2014;48:304–13.

37. Schneck CA, Riesco MLG, Bonadio IC, Diniz CSG, Oliveira SMJV. Resultados maternos e neonatais em centro de parto normal peri-hospitalar e hospital. Rev. Saúde Pública. 2012;46:77–86.

38. Silva FMB da, Paixao TCR da, Oliveira SMJV, Leite JS, Riesco MLG, Osava RH. Care in a birth center according to the recommendations of the World Health Organization. Rev. Esc. Enferm. USP. 2013;47:1031–8.

39. Mouta RJO, Progianti JM. Estratégias de luta das enfermeiras da Maternidade Leila Diniz para implantação de um modelo humanizado de assistência ao parto. Texto Contexto - Enferm. 2009;18:731–40.

40. Prata JA, Progianti JM, Pereira AL de FPAL de F. O contexto brasileiro de inserção das enfermeiras na assistência ao parto humanizado. Rev Enferm UERJ. 2012;20:105–10.


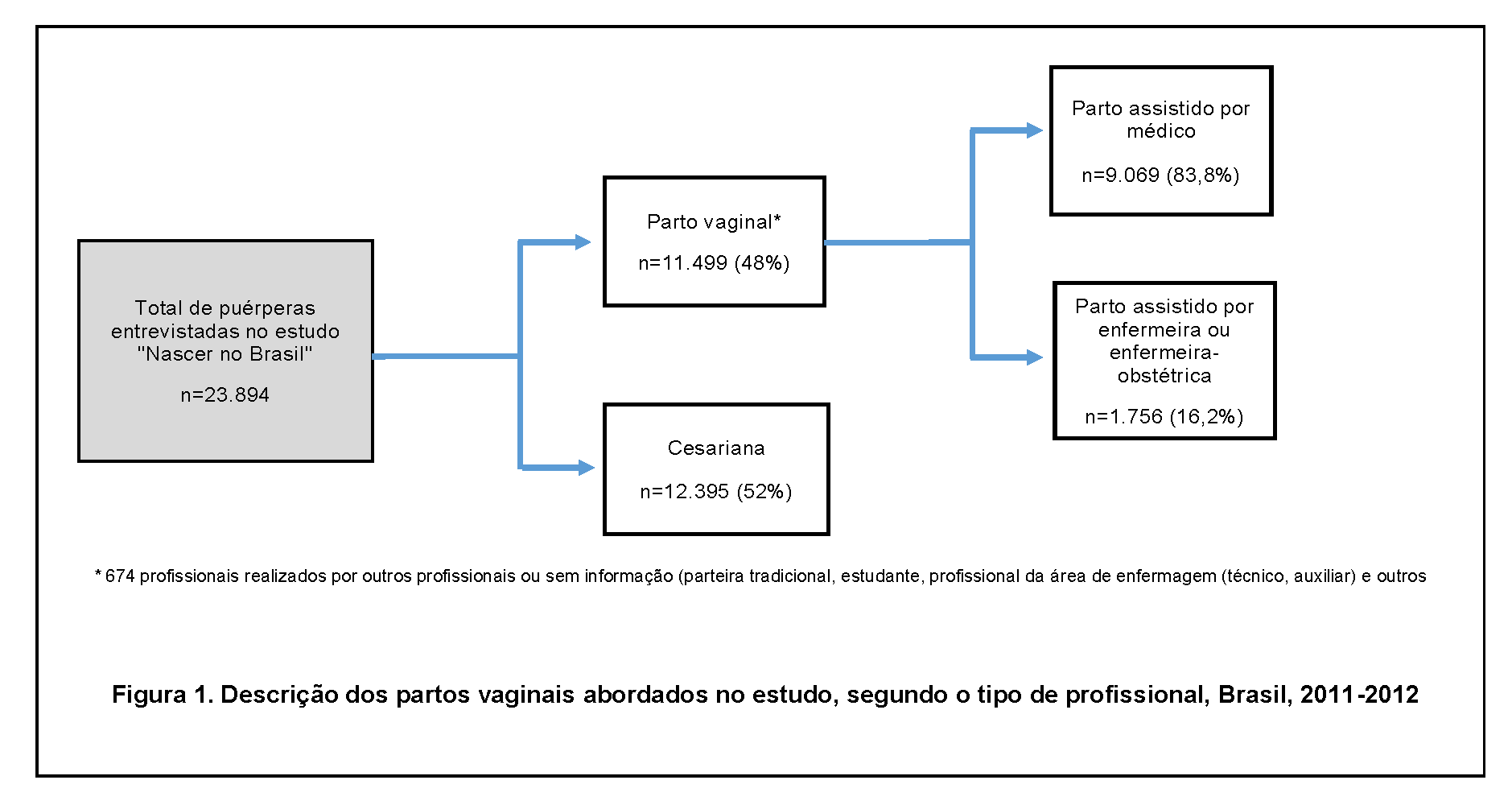


| **Tabela 1** |  |  |  |  |  |
| --- | --- | --- | --- | --- | --- |
| **Frequência de nascimentos segundo o tipo de profissional que assistiu o parto. Brasil, 2011-2012** | | | | | |
|  |  | **Profissional que assistiu o parto (N= 11.499)** | |  |  |
|  |  | **Médico (%)** | **Enfermeira/ Enfermeira-obstétrica (%)** | **p-valor*** |  |
| Região geográfica | Norte | 75,9 | 24,1 | 0,001 |  |
|  | Nordeste | 90,1 | 9,9 |  |
|  | Sudeste | 76,5 | 23,5 |  |
|  | Sul | 92,5 | 7,5 |  |
|  | Centro-Oeste | 99,2 | 0,8 |  |
| Localização | Interior | 83,4 | 16,6 | 0,877 |  |
|  | Capital | 84,3 | 15,7 |  |
| Fonte de pagamento | Pública | 83,5 | 16,5 | 0,054 |  |
|  | Privada | 92,2 | 7,8 |  |
| * p-valor do teste qui-quadrado para comparação entre médicos e enfermeiras/enfermeiras-obstétricas | | | | |  |
|  |  |  |  |  |  |
|  |  |  |  |  |  |
|  |  |  |  |  |  |
|  |  |  |  |  |  |

| **Tabela 2** |  |  |  |  |
| --- | --- | --- | --- | --- |
| **Características das puérperas segundo o tipo de profissional que assistiu o parto. Brasil, 2011-2012** | | | | |
|  |  | **Profissional que assistiu o parto (N= 11.499)** | |  |
|  |  | **Médico (%)** | **Enfermeira/ Enfermeira-obstétrica (%)** | **p-valor*** |
| Idade (anos) | 12 a 19 | 25,5 | 22,4 | 0,040 |
|  | 20 a 34 | 66,8 | 70,0 |
|  | ≥ 35 | 7,7 | 7,6 |
| Cor da pele | Branca | 27,5 | 26,8 | 0,261 |
|  | Preta | 10,5 | 8,3 |
|  | Parda | 60,2 | 63,8 |
|  | Amarela | 1,1 | 0,9 |
|  | Indígena | 0,7 | 0,2 |
| Situação conjugal | Com companheiro | 79,1 | 77,2 | 0,243 |
|  | Sem companheiro | 20,9 | 22,8 |
| Classe econômica | A/B | 14,7 | 13,2 | 0,592 |
|  | C | 54,9 | 57,6 |
|  | D/E | 30,4 | 29,2 |
| Anos de estudo | < 8 | 34,2 | 32,9 | 0,362 |
|  | 8 a 11 | 30,0 | 30,5 |
|  | ≥ 12 | 35,8 | 36,6 |
| Número de partos anteriores | Nulípara | 43,6 | 39,0 | 0,002 |
|  | 1-2 partos | 43,6 | 44,8 |
|  | ≥ 3 partos | 12,8 | 16,2 |
| Risco obstétrico | Baixo | 64,3 | 63,9 | 0,854 |
|  | Alto | 35,7 | 36,1 |
| * p-valor do teste qui-quadrado para comparação entre médicos e enfermeiras/enfermeiras-obstétricas | | | | |

|  | **Médico (%)** | **Enfermeira/ Enfermeira-obstétrica (%)** | **Total (%)** | **OR*** | **IC 95%** |
| --- | --- | --- | --- | --- | --- |
| **Boas práticas no trabalho de parto** |  |  |  |  |  |
| Ingestão de líquidos e alimentos (dieta livre) | 26,1 | 48,7 | 29,8 | 2,35 | 1,62-3,39 |
| Deambulação | 47,9 | 61,1 | 50,1 | 1,74 | 1,29-2,34 |
| Uso de métodos não farmacológicos | 28,7 | 45,1 | 31,3 | 1,87 | 1,29-2,72 |
| Uso de partograma | 51,9 | 68,3 | 54,6 | 1,94 | 1,15-3,29 |
| **Intervenções durante o trabalho de parto** |  |  |  |  |  |
| Uso de cateter venoso | 72,3 | 64,7 | 71,1 | 0,66 | 0,43-1,03 |
| Uso de ocitocina | 47,2 | 47,3 | 47,2 | 0,90 | 0,59-1,37 |
| Amniotomia | 53,9 | 50,6 | 53,3 | 0,83 | 0,57-1,21 |
| Analgesia raquidiana/epidural | 11,1 | 4,8 | 10,1 | 0,29 | 0,12-0,72 |
| **Intervenções durante o parto** |  |  |  |  |  |
| Posição de litotomia | 92,8 | 87,5 | 92,0 | 0,44 | 0,25-0,77 |
| Manobra de Kristeller | 38,7 | 27,2 | 36,8 | 0,56 | 0,41-0,76 |
| Episiotomia | 57,7 | 38,9 | 54,6 | 0,42 | 0,26-0,67 |
| * Modelo ajustado por região geográfica de residência, localização, idade, escolaridade, forma de pagamento do parto e número de partos anteriores | | | | | |
| IC 95%: intervalo de confiança de 95%; OR: razão de chances | |  |  |  |  |

| **Tabela 4** |  |  |  |
| --- | --- | --- | --- |
| **Modelo de regressão logística (OR ajustada) para o uso de boas práticas e intervenções obstétricas de acordo com a** | | | |
| **maternidade que inclui a participação direta de enfermeiras e enfermeiras-obstétricas na assistência ao parto. Brasil, 2011-2012** | | | |
|  | **Maternidade com participação de enfermeiras e enfermeiras-obstétricas na assistência ao parto (N = 84)** | | |
|  | **Total (%)** | **OR*** | **IC 95%** |
| **Boas práticas no trabalho de parto** |  |  |  |
| Ingestão de líquidos e alimentos (dieta livre) | 33,9 | 2,24 | 1,61-3,12 |
| Deambulação | 57,0 | 1,73 | 1,32-2,27 |
| Uso de métodos não farmacológicos | 34,3 | 2,09 | 1,57-2,79 |
| Uso de partograma | 49,0 | 1,85 | 1,15-2,96 |
| **Intervenções durante o trabalho de parto** |  |  |  |
| Uso de cateter venoso | 67,5 | 0,74 | 0,54-1,03 |
| Uso de ocitocina | 44,1 | 0,93 | 0,69-1,24 |
| Amniotomia | 49,9 | 0,70 | 0,52-0,94 |
| Analgesia raquidiana/epidural | 4,7 | 0,36 | 0,14-0,91 |
| **Intervenções durante o parto** |  |  |  |
| Posição de litotomia | 92,6 | 1,04 | 0,48-2,23 |
| Manobra de Kristeller | 33,6 | 0,65 | 0,51-0,82 |
| Episiotomia | 47,1 | 0,54 | 0,37-0,79 |
| Cesariana | 41,4 | 0,78 | 0,62-0,98 |
| * Modelo ajustado por região geográfica de residência, localização, idade, escolaridade, forma de pagamento do parto e número de partos anteriores | | | |
| IC 95%: intervalo de confiança de 95%; OR: razão de chances | |  |  |
