# Supplementary material for: Labor and birth care by nurse with midwifery skills in Brazil
Source: Reprod Health. 2016 Oct 17;13(Suppl 3):123. doi: 10.1186/s12978-016-0236-7 (PMC5073910; doi:10.1186/s12978-016-0236-7)
Supplement: Additional file 2: — Peer review. (DOC 57 kb) [file 12978_2016_236_MOESM2_ESM.doc]

**Referee’s comments to the authors– this sheet WILL be seen by the author(s) and published with the article**

| Title | Labor and birth care by nurses with midwifery skills in Brazil |
| --- | --- |
| Author(s) | Silvana Granado Nogueira da Gama, Jacqueline Alves Torres, Odaléa Maria Bruggemann, Maria Helena da Silva Bastos, Elaine Fernandes Viellas de Oliveira, Arthur Orlando Corrêa Schiller and Maria do Carmo Leal |
| Referee’s name | Nadia Narchi |

**When assessing the work, please consider the following points, where applicable:**

1. Is the question posed by the authors new and well defined? Yes
2. Are the methods appropriate and well described, and are sufficient details provided to replicate the work? Yes
3. Are the data sound and well controlled? Yes
4. Does the manuscript adhere to the relevant standards for reporting and data deposition? No
5. Are the discussion and conclusions well balanced and adequately supported by the data? No
6. Do the title and abstract accurately convey what has been found? No
7. Is the writing acceptable? No

Please make your report as constructive and detailed as possible in your comments so that authors have the opportunity to overcome any serious deficiencies that you find and please also divide your comments into the following categories:

- Major Compulsory Revisions (which the author must respond to before a decision on publication can be reached)
- Minor Essential Revisions (such as missing labels on figures, or the wrong use of a term, which the author can be trusted to correct)
- Discretionary Revisions (which are recommendations for improvement but which the author can choose to ignore)

Where possible please supply references to substantiate your comments.

When referring to the manuscript please provide specific page and paragraph citations where appropriate.

**General comments:**

The work is relevant and important to the Brazilian context that requires a large number of **nurse midwives** and **midwives** (both nursing professions in Brazil) to change the midwifery care model with consequent reduction in cesarean section rates and unnecessary intervention rates during labor and birth.

**Major compulsory revisions:**

The approach of the introduction is limited because it excludes information on how the training and the work of nurse midwives and midwives are organized in the country, both at present and in the past. The references used discuss the importance of the training and the work of midwives, not of nurses. It´s important and relevant to present the reasons that led the country to have this situation. It´s not true that since 1986 nurse midwives have been legally recognized as part of the pregnancy and delivery care. Much long before 1986 nurses, nurse midwives and midwives already legally worked in the midwifery care (see law and nursing/midwifery history in Brazil).

The penultimate paragraph of the discussion section states that there is only one program, nationally, that offers a degree in nurse midwifery. It´s not true: there is only one program, nationally, that offers direct-entry degree in midwifery; there is many programs, nationally, that offers specialization in midwifery for nurses.

The last paragraph of the conclusion section states that the lower caesarean section rates may be the result of greater management participation only of nurses. This statement disregards the existence of direct-entry midwives in the country which is unacceptable.

M**inor essential revisions:**

**Discretionary revisions:**

*(continue on the next sheet)*

**Referee’s comments to the authors– this sheet WILL be seen by the author(s) and published with the article**

| Title | Labor and birth care by nurses with midwifery skills in Brazil |
| --- | --- |
| Author(s) | Silvana Granado Nogueira da Gama, Jacqueline Alves Torres, Odaléa Maria Bruggemann, Maria Helena da Silva Bastos, Elaine Fernandes Viellas de Oliveira, Arthur Orlando Corrêa Schiller and Maria do Carmo Leal |
| Referee’s name | Christine McCourt |

**When assessing the work, please consider the following points, where applicable:**

1. Is the question posed by the authors new and well defined?
2. Are the methods appropriate and well described, and are sufficient details provided to replicate the work?
3. Are the data sound and well controlled?
4. Does the manuscript adhere to the relevant standards for reporting and data deposition?
5. Are the discussion and conclusions well balanced and adequately supported by the data?
6. Do the title and abstract accurately convey what has been found?
7. Is the writing acceptable?

Please make your report as constructive and detailed as possible in your comments so that authors have the opportunity to overcome any serious deficiencies that you find and please also divide your comments into the following categories:

- Major Compulsory Revisions (which the author must respond to before a decision on publication can be reached)
- Minor Essential Revisions (such as missing labels on figures, or the wrong use of a term, which the author can be trusted to correct)
- Discretionary Revisions (which are recommendations for improvement but which the author can choose to ignore)

Where possible please supply references to substantiate your comments.

When referring to the manuscript please provide specific page and paragraph citations where appropriate.

**General comments:**

This is a sound piece of work and the research is important to publish. However, the article is not currently of publishable quality and needs careful attention to definitions and meanings.

There is some lack of clarity in the text at a number of points, probably owing to use of English as a second language but also need for greater clarity about definitions, particularly for an international audience. See detailed recommendations below.

**Major compulsory revisions:**

Terminology: This is an international journal so care with terminology and working definition is needed. In the background, for example, it states that care by nurse midwives is recommended by WHO but that is not accurate. Care by midwives or nurses with midwifery skills is recommended. Even in Brazil, given that there is a direct entry programme at USP, care may be theoretically by midwives. For further clarity in the text it may be worth explaining the Brazilian terminology and the best available English translations such as midwives (parteiras profisionais) or by nurse midwives (enfermeira ostetrica). This text therefore needs to be corrected.

Similarly, in the next paragraph, the terminology is not accurate regarding the Lancet series, which does not specify nurse midwives. It would be more accurate to use the term midwives, having previously explained definitions. So, this should state instead: ‘show that midwifery, especially when offered in collaboration with physicians and other team members, is safe…’

**Discretionary revisions:**

*(continue on the next sheet)*

*Continued*:

Likewise, below this, in the description of the Brazil setting, for international translation accuracy, this should say ‘training in midwifery’ not training in nurse-midwifery. The ICM definition of a midwife is the key reference point.

A clear definition is essential as this underpins the analysis, and again, for international readers, it is important to know what the range of roles (and related training) is in this study and involved in the analysis. To simply say nurses, as in your statement of the aim, is too confusing, as in some countries this would imply people with no midwifery training. Please clarify who was included in your analysis.

**Methods**

The following is not clear: ‘The second sample was composed of the number of days of hospital stay (minimum of 7 days for each hospital),’ so please restate.

Definiton of low obstetric risk is not clear is some aspects: ‘and gave birth vaginally with cephalic presentation at term.’ Does this mean in a previous pregnancy? If so, are all primps excluded from being categorized as low risk?

**Findings**

The following sentence is confusing and needs correction: ‘The use of a partogram, which is recommended for accompanying the evolution of labor, was rarely utilized’

Suggested amendment: ‘The use of a partogram, which is recommended for assessing and recording the progress of labor, was limited..’

For clarity, the sentence: ‘Of the 266 sites studied, less than one-third (N=84) had a delivery attended to by a nurse during data collection’ should be amended to: Of the 266 sites studied, less than one-third (N=84) had a delivery attended to by a nurse during the data collection period’

**Discussion**

The meaning of the following would work better if the text were reversed, from: ‘ Physicians as well as nurses exposed the women…’ to ‘nurses, as well as physicians, exposed the women..’

The following statement does not make sense in terms of the clinical evidence, but I think again this is a use of English issue: ‘Despite much evidence that shows that the use of oxytocin should be limited to reducing the time of labor’ – please restate.

The following: ‘Kristeller maneuver; practices that can cause discomfort and pain [34]’ is not accurate in terms of the clinical evidence. It should state that these can also lead to worse outcomes and pose risks for women – this is particularly the case for Kriteller, which is not allowed in many countries on the basis of risks to the mother. It is not merely a matter of pain and discomfort but more serious than this, in both cases.

What are ‘Normal Labor Centers’ – please define

Continued:

The following text would be more useful in the background:

In Brazil, the inclusion of a nursing professional in delivery care is occurring slowly, and for this reason the present study examined the participation of all nurses, regardless of specialization. Brazilian nurses receive general training. After graduation, there is the possibility of specialization for those who desire further training in nurse midwifery. There is only one program, nationally, that offers a degree in nurse midwifery.

You can then comment on the implications of the study for this situation in the discussion.

I felt the conclusion could be developed a little more in terms of implications.

**General comments on the text**

**Abstract**

The following sentence is unclear, so please rephrase ‘For the use of technological analysis related to postpartum women according to the hospital where deliveries occurred, hospitals where nurses were the providers of delivery care were considered as the unit of analysis.’

The following sentence would also be better rephrased, as follows:

‘A logistic regression model was applied to examine association between the use of good practices and interventions during labor and delivery and the type of provider.’

The next sentence (and also in the method section) is also confusing as the usage of assistive is not appropriate – it has a different specific meaning in English from the one intended. Suggest amendment to:

**‘**Another model tested the association between use of care practices during labor and the type of care team.’

In the conclusions the following sentence is also misleading and suggest you rephrase it as follows:

‘It may be easier to introduce new approaches rather than to eliminate old ones.’

Keywords: suggest you include the keyword midwifery as although in Brazil these are nurses, they are nurses using midwifery skills.

Terms – it is generally accepted as preferable to use the term ‘births’ rather than ‘deliveries’

# Supplement Editor comments

Please pay special attention to the concerns raised about terminology and the need that this terminology could be interpreted at an international level and readers.
